# The Role of Sulfhydryl (Thiols) Groups in Oral and Periodontal Diseases

**DOI:** 10.3390/biomedicines12040882

**Published:** 2024-04-16

**Authors:** Sabetim Cerkezi, Marija Nakova, Icko Gorgoski, Kenan Ferati, Arberesha Bexheti-Ferati, Andrea Palermo, Alessio Danilo Inchingolo, Laura Ferrante, Angelo Michele Inchingolo, Francesco Inchingolo, Gianna Dipalma

**Affiliations:** 1 Orthodontic Department, Dentristy School, Medical Science Faculty, State University of Tetova, 1220 Tetova, North Macedonia; sabetim.cerkezi@unite.edu.mk; 2 Periodontology Department, Dentistry School, Medical Science Faculty, State University of Tetova, 1220 Tetova, North Macedonia; marija.nakova@ibu.edu.mk; 3 Faculty of Natural Sciences and Mathematics, University St. Cyril and Methodius, 1000 Skopje, North Macedonia; icko@pmf.ukim.mk; 4 Faculty of Medicine, State University of Tetova, 1220 Tetovo, North Macedonia; kenan.ferati@unite.edu.mk (K.F.); ar-beresha.ferati@unite.edu.mk (A.B.-F.); 5 College of Medicine and Dentistry, Birmingham B4 6BN, UK; andrea.palermo2004@libero.it; 6 Department of Interdisciplinary Medicine, University of Bari “Aldo Moro”, 70121 Bari, Italy; ad.inchingolo@libero.it (A.D.I.); lauraferrante79@virgilio.it (L.F.); giannadipalma@tiscali.it (G.D.)

**Keywords:** free radicals, antioxidants, gingivitis, periodontitis

## Abstract

Aim. The sulfhydryl (thiols) group of glutathione plays an important role in the neutralization of foreign organic compounds and the reduction in peroxides. The purpose of the study is to evaluate the concentration of sulfhydryl groups in the gingival tissue of healthy individuals and those with gingivitis or periodontitis, and to examine the differences between these groups. Material and methods. To assess the concentration of sulfhydryl groups (thiols) in the gingival tissue of healthy individuals and those with gingivitis or periodontitis, we used spectrophotometric analysis using dithionitrobenzoate (DTNB) as a reagent to measure the accessible sulfhydryl groups present in gingival tissue proteins. The sample was divided into three distinct groups: individuals with periodontal health, gingivitis, and periodontitis, and different indices were used to assess the periodontal status of the participants. Next, a statistical analysis was conducted to compare the concentrations of sulfhydryl groups among the different groups of patients. Conclusions. The results of this study showed significantly decreased levels of sulfhydryl (thiols) groups in gingival tissue from patients with gingivitis and periodontitis, compared with healthy people (control group). These results confirm the role of sulfhydryl (thiols) groups in defense against free radicals. They share a significant role in detoxification, signal transduction, apoptosis, and various other functions at the molecular level.

## 1. Introduction

Periodontal disease represents a broad spectrum of disorders involving periodontal tissues [1]. The two main forms, gingivitis and periodontitis, are often caused by the accumulation of biofilm in dental plaque [2,3]. Gingivitis is an inflammatory condition limited to the gums, but can, in predisposed subjects, evolve towards the more severe and destructive periodontitis [4,5]. The epithelium of the ulcerated periodontal pocket can constitute a direct route for the entry of periodontal pathogens, predominantly Gram-negative anaerobes (*Actinobacillus actinomycetemcomitans*, *Porphyromonas gingivalis*, *Tannerella forsythia*, *Fusobacterium nucleatum*, *Prevotella intermedia*), which colonize the subgingival area and pass into the systemic circulation, potentially affecting other organs [6,7,8,9]. The organism triggers a host response through the establishment of an inflammatory reaction, and responds to biofilm accumulation in dental plaque through the establishment of an inflammatory reaction [10,11,12,13,14,15,16]. Dental biofilm is a complex microbial community that forms on the surface of teeth and can be composed of bacteria, fungi, and other microorganisms. This biofilm, along with other risk factors such as unhealthy lifestyles, genetic predisposition, and environmental factors, plays a key role in the development and progression of periodontitis [10,17,18,19]. Over the years, our understanding of this process has grown steadily, although at times there have been conflicting results in the scientific evidence. For example, it has been observed that ecological stress can affect the composition of the oral microbiota, promoting the growth of pathogenic [20,21]. These bacteria can proliferate and cause damage to gingival tissues, triggering an inflammatory response.

Neutrophils play a crucial role in the body’s inflammatory response. They are white blood cells that are rapidly recruited to damaged tissues to fight harmful pathogens [22,23,24]. When inflammation occurs, neutrophils are activated and migrate to the site of infection or tissue damage. This process, known as neutrophilia, is a hallmark of acute inflammation and is an important mechanism of the body’s defense against infection.

In inflamed periodontal tissues, neutrophils play a key role in eliminating pathogenic bacteria and promoting healing of damaged tissues. They release enzymes and chemicals that destroy bacteria and contribute to the removal of damaged tissues. However, excessive neutrophil activation and chronic inflammation can lead to damage to surrounding tissues and contribute to the progression of periodontal disease.

**Figure 1:**
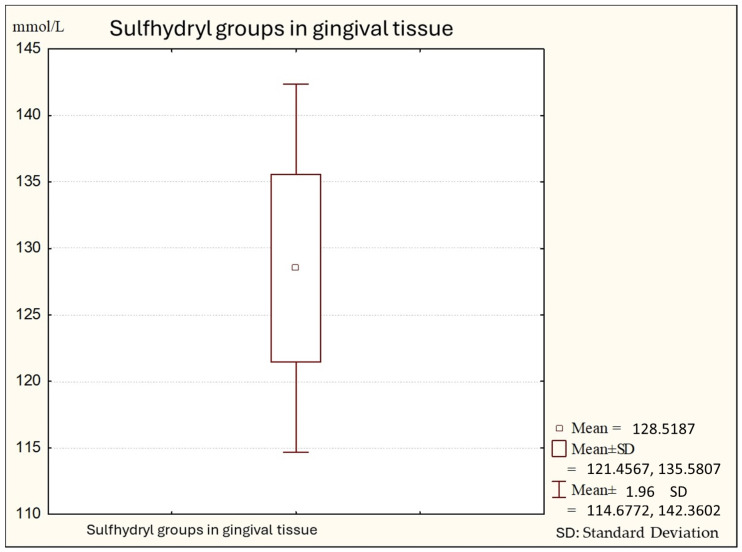
Descriptive statistics of sulfhydryl group in the gingival tissue from healthy people (Group A).

In addition, it is important to consider the role of inflammatory mediators produced by neutrophils and other immune cells in the context of periodontitis. These mediators, such as proinflammatory cytokines and tumor necrosis factors, may contribute to the progression of inflammation and destruction of periodontal tissues. Therefore, understanding the role of neutrophils and inflammatory mediators in the pathogenesis of periodontitis is critical for the development of new therapeutic strategies aimed at controlling inflammation and promoting gingival tissue healing [25]. While the body normally produces ROS during cellular processes, excessive ROS production due to factors like stress or pollution can lead to oxidative stress, causing cellular damage [10,26,27,28,29,30,31,32,33,34,35,36,37,38,39,40,41]. Neutrophils release inflammatory mediators and reactive oxygen species (ROS), which are highly reactive molecules containing oxygen [42,43,44,45,46,47,48,49,50]. ROS, including free radicals, are unstable and constantly seek to stabilize themselves by interacting with nearby molecules to overcome the body’s natural defenses and lead to cellular damage, causing oxidative stress (Figure 1).

This oxidative stress is associated with many diseases, from premature aging to more serious conditions such as heart disease, cancer, and neurodegenerative diseases [51,52,53]. ROS include oxygen-derived free radicals such as superoxide, hydroxyl, nitric oxide, hydrogen peroxide, and hypochlorous acid [54,55]. These molecules emerge in inflamed tissues and can cause damage to lipids, proteins, and deoxyribonucleic acid, eventually leading to tissue degradation. This phenomenon is believed to underlie inflammatory conditions affecting the periodontium, evident as gingivitis and periodontitis [56,57].

The primary (nicotinamine adenine dinucleotide) targets of highly reactive ROS are DNA, RNA, proteins, and lipids. Most damage stems from the hydroxyl radical generated through the Fenton reaction, reliant on iron or other divalent metal ions, along with a source of reducing equivalents, potentially NADH, to regenerate the metals, as detailed by Stadtman [58,59,60]. At a cellular level, exposure of proteins to ROS results in modifications of amino acid side chains, consequently altering protein structure and leading to functional disturbances within cellular metabolism [61]. Among the 20 amino acids constituting proteins, several are susceptible to direct modification via side chain reactions with ROS. Predominantly, those containing sulfhydryl groups (thiol groups) and those with aromatic side chain groups are affected.

Sulfhydryl groups show significant and diverse reactivity, easily oxidizing to form disulfides, or sulfenic or sulfonic acids, and easily undergoing processes such as alkylation, acylation, and thiol–disulfide exchange [62,63]. They react with heavy metal ions to form mercaptides and with aldehydes and ketones to form mercaptals and mercaptols, respectively. These groups play crucial roles in biochemical processes: the sulfhydryl group of coenzyme A, lipoic acid, and 4-phosphopantotheine participates in enzymatic reactions for the formation and transfer of acyl residues, which are essential for lipid and glucose metabolism. The sulfhydryl group of glutathione is critical in neutralizing foreign organic compounds, reducing peroxides, and acting as a coenzyme [64,65,66]. In addition, cysteine residues in proteins participate in catalytic effects, binding to substrates, and binding of coenzymes and metal ions. Enzymatic sulfhydryl groups catalyze the formation of intermediates with substrates or their residues, while in oxidative enzymes they facilitate electron and proton transfers from substrates to acceptors [67,68,69,70]. These reactions are critical for many metabolic pathways and biochemical processes crucial to cellular life. For example, the sulfhydryl group present in coenzyme A is essential for the synthesis and transport of fatty acids, while glutathione, with its sulfhydryl group, plays a key role in cell defense against oxidative stress and detoxification of foreign compounds. In addition, the sulfhydryl groups of proteins may be involved in the regulation of enzyme activity and cellular signal transduction, thus contributing to the overall functioning of the cell and the organism.

Blocking sulfhydryl groups results in partial or complete inhibition of the activity of many enzymes. Disulfide bonds (S–S), formed during protein biosynthesis through the oxidation of sulfhydryl groups, play a crucial role in stabilizing protein structures, including enzymes, antibodies, and various hormones [71,72]. Disruption of disulfide bonds leads to the denaturation of protein structures and the loss of biological activity [73,74,75,76]. These bonds significantly contribute to the tertiary structure of proteins.

Chemical and histochemical studies on epithelium suggest the potential significance of sulfhydryl and disulfide groups in gingival metabolism [77,78,79,80]. 

Antioxidants are a group of substances that prevent the oxidation of substrates by ROS, offering protection against oxidative stress. The thiol antioxidants include reduced glutathione, glutaredoxin, and N-acetyl cysteine [81,82,83]. Thiols are integral to the intraprotein structure and exist in equilibrium with disulfide groups. They prevent the irreversible unfolding of protein structures due to oxidative stress. Among the body’s antioxidants, thiols constitute a major portion and play a crucial role in defending against reactive oxygen species. Total thiols comprise intracellular and extracellular forms, existing as free or bound to proteins, including oxidized or reduced glutathione. Among protein-bound thiols, albumin predominantly binds to the sulfhydryl group at its cysteine-34 portion. Thiols contribute significantly to defense against free radicals, involving detoxification, signal transduction, apoptosis, and various molecular-level functions [84,85], especially in the detection of low-molecular-weight thiols in gingival cervical fluid (GCF) as a defense against ROS-mediated damage.

Antioxidants, thus, are molecules that counteract the harmful action of free radicals and ROS, helping to protect cells from oxidative damage [86,87,88]. In periodontitis, where oxidative stress can play a significant role in inflammation and disease progression, antioxidants can play an important role in reducing these harmful effects because they help neutralize ROS, thus reducing oxidative stress in inflamed gum tissue and limiting inflammation and associated tissue damage [89,90,91]. They also influence the inflammatory mediators involved in periodontitis, helping to regulate the inflammatory response and limiting the worsening of the condition. This happens through various mechanisms [92,93]:

1. Neutralizing free radicals that activate the inflammatory cascade, stimulating the production of inflammatory mediators such as proinflammatory cytokines (such as TNF-α and IL-1β) [2,94] and tumor necrosis factors. By neutralizing ROS, antioxidants can reduce this inflammatory response [95,96];
2. By inhibiting intracellular signaling pathways involved in the inflammatory response. They can block the activation of certain transcription factors that regulate the gene expression of inflammatory mediators, thus reducing their production [97,98,99];
3. By modulating the activity of cells involved in the inflammatory response such as macrophages and lymphocytes, reducing the production of proinflammatory cytokines, and, instead, increasing the production of anti-inflammatory molecules [100,101];
4. Reducing oxidative stress, and therefore ROS levels, which support the inflammatory response [102].

Antioxidants exert a regulatory effect on the inflammatory response in periodontitis, helping to limit the worsening of the condition and promoting a more favorable environment for the healing of inflamed tissues [103]. However, it is important to emphasize that understanding the specific mechanisms involved requires further research to more precisely define the role of antioxidants in the management of periodontitis [104,105,106,107]. They support the healing of damaged gingival tissues, facilitating repair and recovery [108,109,110]. In addition to antioxidants, some natural substances have been studied for their effectiveness in managing periodontitis and reducing gingival inflammation; coenzyme Q10, vitamin C, tea tree essential oil, aloe vera, phenols, turmeric, and ginger [62]. The mechanisms by which antioxidants exert their beneficial effects on periodontitis are multiple and complex. Antioxidants work by neutralizing free radicals and reducing oxidative stress in inflamed tissues. This process helps limit cellular damage and preserve the structural integrity of tissues, thereby promoting healing. In addition, antioxidants can modulate gene expression and inflammatory cytokine production, thereby influencing the immune and inflammatory response in the context of periodontitis.

Coenzyme Q10, for example, is involved in cellular energy production processes and has demonstrated antioxidant and anti-inflammatory properties that may contribute to improved periodontal health. Vitamin C is known for its role in collagen synthesis and wound healing, as well as acting as a powerful antioxidant. Tea tree essential oil has demonstrated antimicrobial and anti-inflammatory activity, which may help control the infection and inflammation associated with periodontitis. Aloe vera is known for its soothing and healing properties, which may promote the healing of inflamed gum tissue. Phenols, found, for example, in green tea, have demonstrated antioxidant and anti-inflammatory properties that may help control gingival inflammation. Turmeric and ginger, on the other hand, have been studied for their potential anti-inflammatory and antioxidant properties, which could be useful in the treatment of periodontitis.

In addition, antioxidants may interact with other treatments used for the management of periodontitis, enhancing their effectiveness and reducing their side effects. For example, the combined use of antioxidants and antibiotic treatments may help reduce bacterial load in inflamed tissues and improve clinical outcomes of treatment. However, further research is needed to fully understand the role of antioxidants in the management of periodontitis and to identify the best therapeutic strategies based on these compounds.

These natural substances can be considered as adjuncts to conventional therapy for periodontitis. However, studies on the relationship between antioxidant status and periodontitis have produced discordant results [111,112,113]: not all studies have observed significant differences in total antioxidant levels between saliva samples from healthy subjects and those with periodontitis. This study aims to further investigate the role of sulfhydryl groups (thiols) in the periodontal environment by exploring their variations in the initial and clinical manifestations of periodontal disease [7,114]. Ultimately, the study of sulfhydryl groups in the periodontal environment represents a promising area of research that could lead to new insights into the pathogenesis and management of periodontal disease. Understanding the molecular mechanisms involved in the physiological and pathological processes of periodontal tissue could pave the way for new diagnostic, therapeutic, and preventive strategies aimed at improving periodontal health and patients’ quality of life.

## 2. Materials and Methods

This research encompassed a cohort of 90 individuals aged between 18 and 64 years. Assessment of the subjects’ periodontal status involved evaluating the Plaque Index (PI) developed by Silness and Loe in 1964 [115,116] and the Gingival Index (GI) devised by Loe and Silness in 1963 [115,117,118], as well as conducting measurements of pocket probing depths and clinical attachment levels. Table 1 shows how 90 participants were divided into three groups according to certain criteria: those with gingivitis (Group B), those with periodontal health (Group A), and those with a periodontitis diagnosis (Group C). [119,120].

According to the stratification and degree of periodontitis in the new classification, the three groups of patients were divided into:

Group A—Individuals with periodontal health (periodontal health): absence of periodontitis;

Group B—Patients with gingivitis (gingivitis): incipient and moderate periodontitis;

Group C—Patients diagnosed with periodontitis (periodontitis): severe periodontitis and advanced periodontitis.

To analyze the biochemical composition and molecular characteristics of gingival tissue, including levels of sulfhydryls, which are important for understanding the redox state and antioxidant activity of gingival tissue in healthy conditions and periodontal disease, marginal and interproximal gingival tissue from all oral cavity districts, taken during gingival biopsy procedures, was used.

The quantification of sulfhydryl (thiol) groups within gingival tissue was conducted using a spectrophotometric method employing dithionitrobenzoic (DTNB) [93,121]. This compound reacts with accessible sulfhydryl (thiol) groups present in proteins, reducing them to stable intermediate compounds known as mixed disulfides, specifically protein S–S compounds, denoted as 5-mercapto-2-nitrobenzoate (MNB) [21,122]. The measurement of MNB, taken after 5 min at 412 nm using a Spectronic 10 UV spectrophotometer (Rochester, NY, USA), was compared against a glutathione standard and quantified in terms of micromoles per liter, following the method outlined by Mochink [123,124,125].

Statistical analysis was performed using the SPSS 7.1 statistical software (SPSS statistical software version 7.1, Chicago, IL, USA) for data processing and analysis [126,127]. The normality of the distribution was determined using the Shapiro–Wilk test, the significance of the differences was estimated with the student’s *t*-test, and differences were taken to be significant at *p* < 0.05.

## 3. Results

Figure 1 shows the average values of sulfhydryl groups (thiol) in gingival tissue from healthy subjects. The average value in this subject is 147.74 (minimal values: 121.00; maximal: 173.00), with a standard deviation of 11.28.

**Figure 2:**
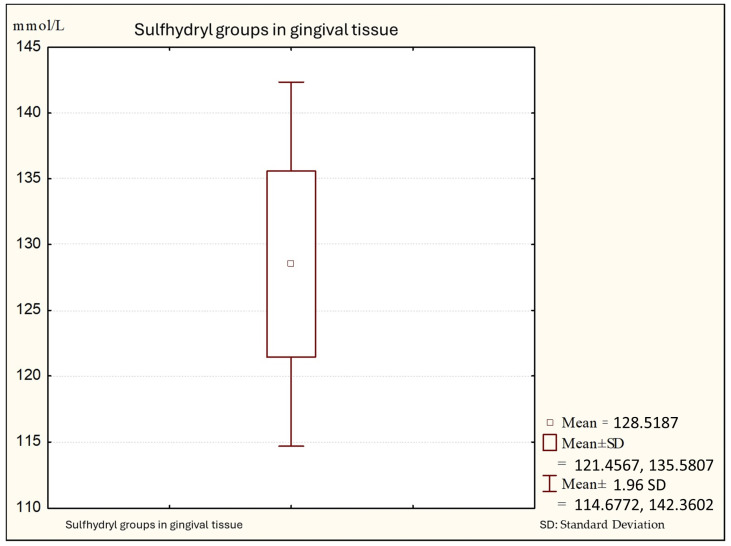
Descriptive statistics of sulfhydryl group in the gingival tissue from people with gingivitis (Group B).

Figure 2 shows that the average values of sulfhydryl groups (thiol) in gingival tissue from patients with gingivitis are 128.88 (minimal values: 110.14; maximal: 140.00 +95%), with a standard deviation of 7.06.

**Figure 3:**
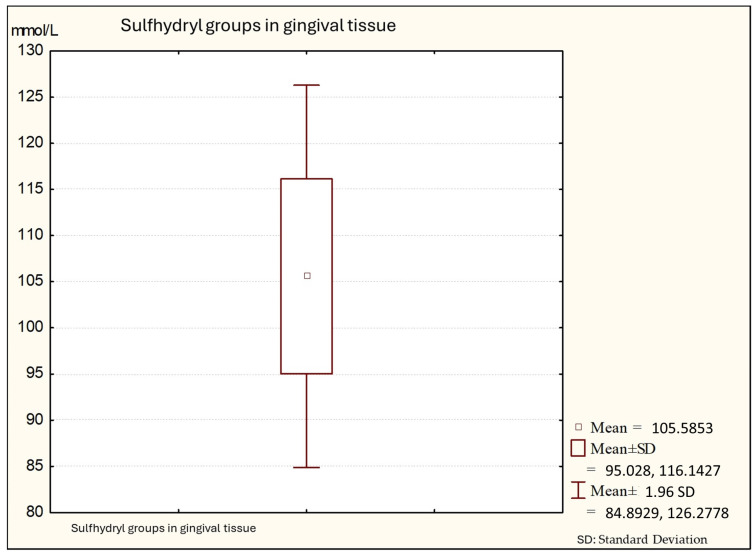
Descriptive statistics of sulfhydryl group in the gingival tissue from people with periodontitis (Group C).

Figure 3 shows that the average values of sulfhydryl groups (thiol) in gingival tissue from patients with periodontitis is 101.64 (minimal values: 91.34; maximal: 130.14), with a standard deviation of 10.56. 

The average values of sulfhydryl are summarized in Table 2.

Table 3 and Table 4 show the concentration, in mmol/L, of the sulfhydryl (thiol) group in gingival tissue, between examined groups (healthy, gingivitis, and periodontitis).

Table 4 is a representation of the results of the statistical analysis in which the three groups were compared. “M = 105.59, M = 128.52, M = 147.74” means were calculated for each group. The numbers in the table, under the letters A, B, and C, are the results of the significance values (*p*-value) of the analysis, which, in this context, are all zero (“0.000”). 

The sulfhydryl (thiol) group concentrations differed significantly across groups, with healthy individuals (control Group A) exhibiting the highest mean value (x = 147.74 mmol/L), followed by individuals with gingival inflammation (Group B) (x = 128.52 mmol/L), and the lowest levels observed in patients with periodontal disease (Group C) (x = 105.59 mmol/L) (*p* < 0.001). Additionally, post hoc analysis revealed statistically significant differences in sulfhydryl group concentrations among all groups (F = 138.91; *p* < 0.001). 

The average concentration of sulfhydryl groups (thiols) was significantly different among patient groups. The highest concentration was observed in subjects with periodontal health, followed by those with gingivitis, and then those with periodontitis. This difference was evaluated as statistically significant (F = 138.91, *p* < 0.001).

## 4. Discussion

Healthy individuals (Group A) exhibited the highest average sulfhydryl group concentration (147.74 mmol/L), significantly higher than both gingivitis (Group B) and periodontitis (Group C) patients.

Gingivitis patients (Group B) showed an intermediate average sulfhydryl group concentration (128.52 mmol/L), significantly lower than healthy individuals (Group A) but higher than periodontitis patients (Group C).

Periodontitis patients (Group C) displayed the lowest average sulfhydryl group concentration (105.59 mmol/L), significantly lower than both healthy individuals (Group A) and gingivitis patients (Group B). A summary of results is provided in Table 5.

**Figure 4:**
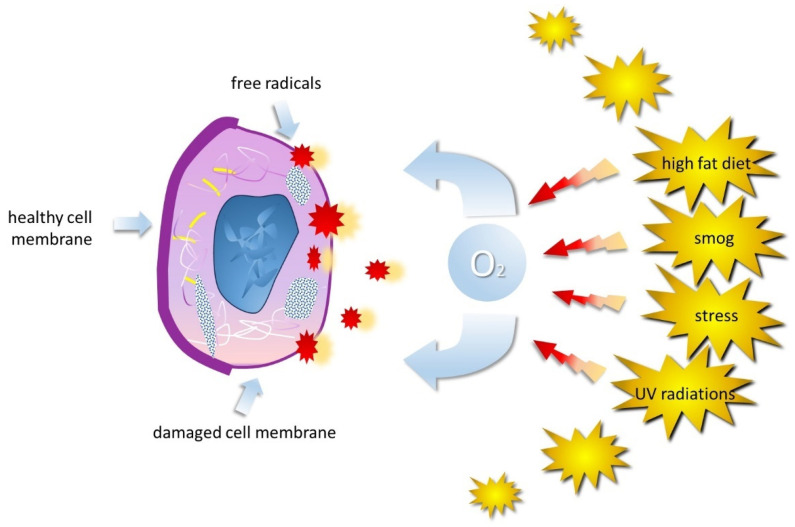
Different physical, chemical, and biological agents can influence the spontaneous generation or physiological production of ROS. These include ionizing and UV radiations, air pollution, various types of pollutants, and foods rich in animal fats, but also aromatic hydrocarbons, pesticides, drugs, bacteria, and stress.

The inflammatory and immune response induced by sub-gingival plaque is the most important factor in the development of this disease [128,129,130]. Development mechanisms of inflammatory-destructive processes in periodontal tissues are not well understood. The disorder is probably multifactorial and it is characterized by the generation of ROS (Figure 4) (Waddington) [129], by activated phagocytes at gingival tissue (Baelum) [131], (Katsuragi H) [132], which can initiate the destruction of connective tissue. Oxidative stress refers to the imbalance due to excess ROS or oxidants over the capability of the cell to mount an effective antioxidant response (Kantarci) [133,134,135,136].

The pathological events which lead to the destruction of the periodontal tissues during gingival inflammation and periodontal destruction have been related to the effect of an imbalance between oxidants and antioxidants in patients with gingivitis and periodontitis (Sakalilioglu) [61,129,137]. In the health of the periodontium, a balance between oxidizing substances (such as free radicals and other reactive molecules) and antioxidants is essential for maintaining a healthy environment [138,139,140]. Gum inflammation and periodontal diseases, such as gingivitis and periodontitis, involve inflammatory processes that can generate excessive free radicals and other oxidizing molecules [141,142]. These molecules, if present in excessive or unbalanced quantities, can damage the periodontal tissues, leading to the destruction of the supporting tissues of the teeth [143,144,145]. An imbalance between these oxidizing and antioxidant substances, which normally protect tissues by neutralizing free radicals and maintaining balance, can promote the progression of gum and periodontal diseases, damaging the tissues and contributing to their destruction [146,147,148].

There are many enzymatic antioxidant defense mechanisms to protect against ROS effects in vivo: SOD, GPx, Cathalasa, uric acid, ascorbate, alpha-tocopherol, urate, thiols, and albumin (Sculley, Nagler, Amerongen) [144,149,150,151]. Chaplle et al. [81,152,153], found lower total antioxidant concentration in the saliva of periodontitis patients when compared to health controls [24,154]. Salivary antioxidant levels (SOD, GPx, reduced GHS, ascorbic acid, alfa tocopherol, were observed to the lower in periodontal patients (Canacki) [146,155,156,157]. In the present study, sulfhydryl (thiol), in gingival tissue, was observed in reduced quantities in the patients with gingivitis and periodontitis (groups B and C), compared with the people from healthy group (Group A) [158,159]. Testing the significance of differences in the concentration of sulfhydryl (thiol) group between the examined groups by post hoc test showed statistically significant differences in the values of this parameter among all groups of patients (F: 138.91; *p* < 0.001; *p* = 0.000) [160]. The lower nonconcentrate of sulfhydryl (thiol group in the gingivitis and periodontitis groups can be attributed to the presence of periodontopathic pathogens that readily degrade them from hydrogen sulfide, which can be toxic [129,158,161]. Similar observations were made by Thomas [162,163], Patel [164], Duarte [165], and Novakovic [166]. They found that gingival tissue analysis revealed that the antioxidative capacity in patients with periodontitis was decreased when compared to the control group [128,167]. This study reported a significant increase in glutathione peroxidase (GSH-Px) and glutathione S-transferase (GST), while a decrease in glutathione (GSH) and total glutathione levels in gingival tissue and serum from an experimental group when compared to the control group [168,169,170]. The ubiquitous tripeptide GSH and other amino thiols are also involved in the production of volatile sulfur responsible for the bad breath in periodontopathic patients (Bald and Glowacki) [171,172,173]. 

The intricate interplay between sulfhydryl (thiol) groups and periodontal health is paramount in understanding the underlying oxidative stress contributing to gingivitis and periodontitis [174,175]. Sulfhydryl groups, specifically within glutathione, serve pivotal roles in neutralizing foreign organic compounds and reducing peroxides [176,177]. They act as significant coenzymes and are present in cysteine residues of amino acids within proteins [142,178]. The blocking or alteration of sulfhydryl groups can lead to considerable enzyme activity inhibition, underscoring their critical importance in protein structure stabilization via disulfide bonds [179,180].

This study yielded compelling evidence demonstrating significantly diminished levels of sulfhydryl (thiol) groups within gingival tissue among patients afflicted with gingivitis and periodontitis when compared to healthy individuals (control group), corroborating the vital role of these groups in combating free radicals [181,182,183]. These findings highlight the pivotal involvement of sulfhydryl groups not only in detoxification processes but also in signal transduction, apoptosis, and various molecular-level functions [184,185,186].

The statistical analyses performed in this study revealed a statistically significant decrease in sulfhydryl (thiol) group concentrations among individuals with gingivitis and periodontitis in comparison to the control group [164,187]. These findings align with previous studies that have emphasized the vital role of antioxidative mechanisms, such as sulfhydryl groups, in mitigating periodontal disease progression [7,188,189]. 

## 5. Conclusions

Oxidative stress in the pathogenesis of gingivitis and periodontitis is the result of an alteration in redox balance and tissues of the innate immunity system. Gingival tissue analysis of the sulfhydryl (thiols) group revealed that the antioxidative activity in patients with gingivitis and periodontitis was decreased when compared to the control group. The observed decline in the antioxidative capacity of sulfhydryl groups in patients with periodontitis compared to the control group suggests a potential avenue for therapeutic intervention. Strategies aimed at restoring or enhancing sulfhydryl group levels within gingival tissues might hold promise as adjunctive therapies in managing and ameliorating the progression of gingivitis and periodontitis. Our study strengthens the growing body of evidence implicating sulfhydryl (thiol) groups in the pathophysiology of periodontal diseases. Further investigations into modulating these antioxidative mechanisms could unveil novel therapeutic avenues to alleviate the burden of these prevalent oral inflammatory conditions. This expanded conclusion aims to encapsulate the significance of sulfhydryl groups, their alterations in periodontal disease, and the potential implications for therapeutic interventions.

## Figures and Tables

**Table 1:** Participants categorized into distinct groups based on specific criteria: health (Group A), gingivitis (Group B), periodontitis (Group C).

90 individuals: - 67 females and 23 males; - The age of the participants ranged from 18 to 64 years; - The samples are of Macedonian origin.	Group A (25 patients): periodontal health.
	Group B (35 patients): gingivitis.
	Group C (30 patients): periodontitis.

**Table 2:** Average values of sulfhydryl group (thiol) in the gingival tissue of healthy persons (Group A), from people with gingivitis (Group B), and from people with periodontitis (Group C).

Parameter	N	Average (mmol/L)	Confidence −95.00% (mmol/L)	Confidence +95.00% (mmol/L)	Min (mmol/L)	Max (mmol/L)	Standard Deviation
Sulfhydryl Group A (thiol) in Gingival tissue	30	147.74	143.52	151.95	121.00	173.00	11.28
Sulfhydryl Group B Gingival tissue	30	128.52	125.88	131.16	110.14	140.00	7.06
Sulfhydryl Group (thiol) C in gingival tissue	30	105.59	101.64	109.53	91.34	130.14	10.56

**Table 3:** Concentration of the sulfhydryl (thiol) group in gingival tissue.

Parameter	SS	Df	MS	SS	df	MS	F	P
Sulfhydryl group	26,719.22	2	13,359.61	8367.04	87	96.17	138.91	0.000

**Table 4:** Concentration of the sulfhydryl (thiol) group in Group A (healthy), Group B (gingivitis), and Group C (periodontitis).

Group	{1} M = 105.59	{2} M = 128.52	{3} M = 147.74
A		0.000	0.000
B	0.000		0.000
C	0.000	0.000	

**Table 5:** Summary of results.

Disease Stage	Average Sulfhydryl Group Concentration (mmol/L)	Confidence Interval (95%)	Min Value	Max Value	Standard Deviation
Healthy (Group A)	147.74	(143.52, 51.95)	121.00	173.00	11.28
Gingivitis (Group B)	128.52	(125.88, 31.16)	110.14	140.00	7.06
Periodontitis (Group C)	105.59	(101.64, 09.53)	91.34	130.14	10.56

Note: Statistical analysis revealed significant differences in sulfhydryl group concentrations among all groups (F = 138.91, *p* < 0.001).

## Data Availability

Data are contained within the article.

## Abbreviations

Deoxyribonucleic acid (DNA), degrees of freedom (Df), dithionitrobenzoic (DTNB), F-statistic (F), gingival cervical fluid (GCF), Gingival Index (GI), glutathione (GSH), glutathione peroxidase (GSH-Px), glutathione s-transferase (GST), interleukin-1β (IL), mean (M), 5-Mercapto-2-Nitrobenzoate (MNB), mean square (MS), nicotinamine adenine dinucleotide (NADH), oxidative stress (OS), P-value (P), Plaque Index (PI), reactive oxygen species (ROS), ribonucleic acid (RNA), sum of squares (SS),tumor necrosis factor-A (TNF-α).
